# A Population-Based Investigation into the Self-Reported Reasons for Sleep Problems

**DOI:** 10.1371/journal.pone.0101368

**Published:** 2014-07-01

**Authors:** David Armstrong, Alex Dregan

**Affiliations:** 1 King's College London, Department of Primary Care and Public Health Sciences, London, United Kingdom; 2 King's College London, Department of Primary Care and Public Health Sciences, London, United Kingdom, and National Institute for Health Research Biomedical Research Centre at Guy's and St Thomas' National Health Service Foundation Trust, London, United Kingdom; Hospital General Dr. Manuel Gea González, Mexico

## Abstract

Typologies of sleep problems have usually relied on identifying underlying causes or symptom clusters. In this study the value of using the patient's own reasons for sleep disturbance are explored. Using secondary data analysis of a nationally representative psychiatric survey the patterning of the various reasons respondents provided for self-reported sleep problems were examined. Over two thirds (69.3%) of respondents could identify a specific reason for their sleep problem with worry (37.9%) and illness (20.1%) representing the most commonly reported reasons. And while women reported more sleep problems for almost every reason compared with men, the patterning of reasons by age showed marked variability. Sleep problem symptoms such as difficulty getting to sleep or waking early also showed variability by different reasons as did the association with major correlates such as worry, depression, anxiety and poor health. While prevalence surveys of ‘insomnia’ or ‘poor sleep’ often assume the identification of an underlying homogeneous construct there may be grounds for recognising the existence of different sleep problem types particularly in the context of the patient's perceived reason for the problem.

## Introduction

In recent years a large number of studies have documented the extensive prevalence of sleep disturbances in the adult population with upwards of 30% considered to be affected [1]. In this burgeoning field there have been debates about how best to define and measure sleep problems but their wide extent has not seemed in doubt. Further, these sleep problems have seemed clearly patterned by age and gender with greater prevalence in the elderly and women [2]–[4]. Such a global picture, however, involves treating sleep problems as homogeneous and, as with any ‘symptom’, this assumption may conceal different underlying ‘types’ of sleep disturbance.

There are a number of ways of disaggregating a symptom complex. The first is to search for different patterns of symptom expression. For sleep disturbance this approach has proved popular in psychiatry where identification of clinical anxiety or depression can be supported by whether sleep disturbance involved difficulty falling asleep or early morning waking. The second method for teasing out sub-types is to search for different underlying causes of the symptom – as when haemoptysis (coughing up blood) was found to be caused by both tuberculosis and lung cancer. This approach has been widely deployed by research which has identified a range of biopsychosocial and socio-economic factors that may help understand differences in sleep problems [4]–[11]; such studies have attempted to develop causal pathways linking external events and experiences with sleep disturbance. But a third approach to understanding the patterning of sleep problems in the population is to classify according to respondents reported own explanation of the problem.

Research which seeks to understand the causes of sleep disturbance attempts to access factors which respondents may not be aware of; indeed, a risk factor for poor sleep (such as gender) may not be part of the respondent's own explanatory framework as it is unlikely people generally account for poor sleep by invoking their gender. Reasons, on the other hand are a central component of a person's explanatory framework and examining the patterning of sleep disturbance by reason provides another way of sub-typing sleep problems which, in their turn, may have important theoretical and practical implications, particularly with respect to prevention and treatment.

The aims of the present study were to identify the different reasons for reporting sleep problems in a nationally representative study of the UK adult population and then to explore how these patient-derived categories were patterned by gender and age, whether there were differences in the way they were experienced and whether they varied by some established predictive factors.

## Materials and Methods

### Participants

The data for the present study is based on 7,403 individuals who participated in the 2007 National Psychiatric Morbidity Survey (NPMS), a randomized cross-sectional survey of adults aged 16 to 74 living in private households in Great Britain. The main aims of the survey were to estimate the prevalence of psychiatric morbidity as well as service usage and health care associated with these disorders among the adult population of Great Britain. In addition, the survey included a variety of lifestyle factors associated with mental disorders. A detailed explanation of the study methodology and design is provided in Singleton et al. [12]. The data used in this study were made available through the UK Data Archive and are freely downloadable upon registration (www.esds.ac.uk).

### Measures

#### Sleep

The sleep problems sub-scale of the Revised Clinical Interview Scale (CIS-R), a structured interview assessing the severity and frequency of psychological symptoms in the week prior to the interview, represented the main source of information on respondents' sleep problems. The subscale includes 10 items referring to a) whether or not respondents had a number of different sleep problems (getting to sleep, waking up earlier than necessary, sleeping more than usual), b) the severity of these problems (how often it happened over the past 7 days, how long trying to get to sleep, how many nights slept more than usual), c) the duration of these problems (varying from less than two weeks to 2 years or more), and d) the main reason for sleep problems. In addition, an overall *sleep problems score* based on five items (sleep problems for ≥4 nights in the past week, spent ≥3 hours on trying to get to sleep on ≥4 nights in the past seven days, slept >3 hours longer than usual on four or more nights in the past week, spent ≥1/4 hour trying to get to sleep on the night with the least sleep, and slept ≥1/4 hour longer than usual on the night with the longest sleep) was available as a measure of sleep problems severity. A score of one was given for a positive answer on any of the five items: values ranging from 0 to 5.

#### Main reason for sleep problems

The information about the main reason for sleep problems was based on an item that provided participants with nine options (noise, shift work/too busy, illness/discomfort, worry/thinking, needing to go to the toilet, having to do something (e.g. look after someone), tired, medication, and other) to select. Participants who were unaware about the reason for their sleep problems were classified as unknown reason.

#### Other measures

In addition to the sleep problem measures several other factors were utilized to explore their association with the main reason for sleep problems. These factors are among the strongest determinants of sleep problems in the literature and are also related to the reasons assessed in the present study [1], [2], [7], [8], [11]. These factors were based on several subscales of the CIS-R and included the overall severity scores for depression, anxiety and worry. Two socio-demographic variables, age (continuous variable) and gender (male/female) were used in the analysis as well as self-reported general health status (a 5-point Likert scale – excellent to poor).

### Data analysis

Descriptive statistical analyses including frequency graphs were initially used to explore the distribution of the respondents' reasons for their sleep problems by gender. Percentage distributions were used to graph the trend of the patterning of sleep reasons by age, sleep behaviour, as well as main correlates of sleep problems (i.e. anxiety, depression, illness). The relationship between the respondents' reasons for sleep problems with specific sleep symptoms, psychiatric morbidity and some known correlates of sleep problems were investigated using chi-square, Fisher's exact test, contingency tables, and logistic regression. All the analyses were conducted using Stata version 10 statistical package.

## Results

**Figure 1:**
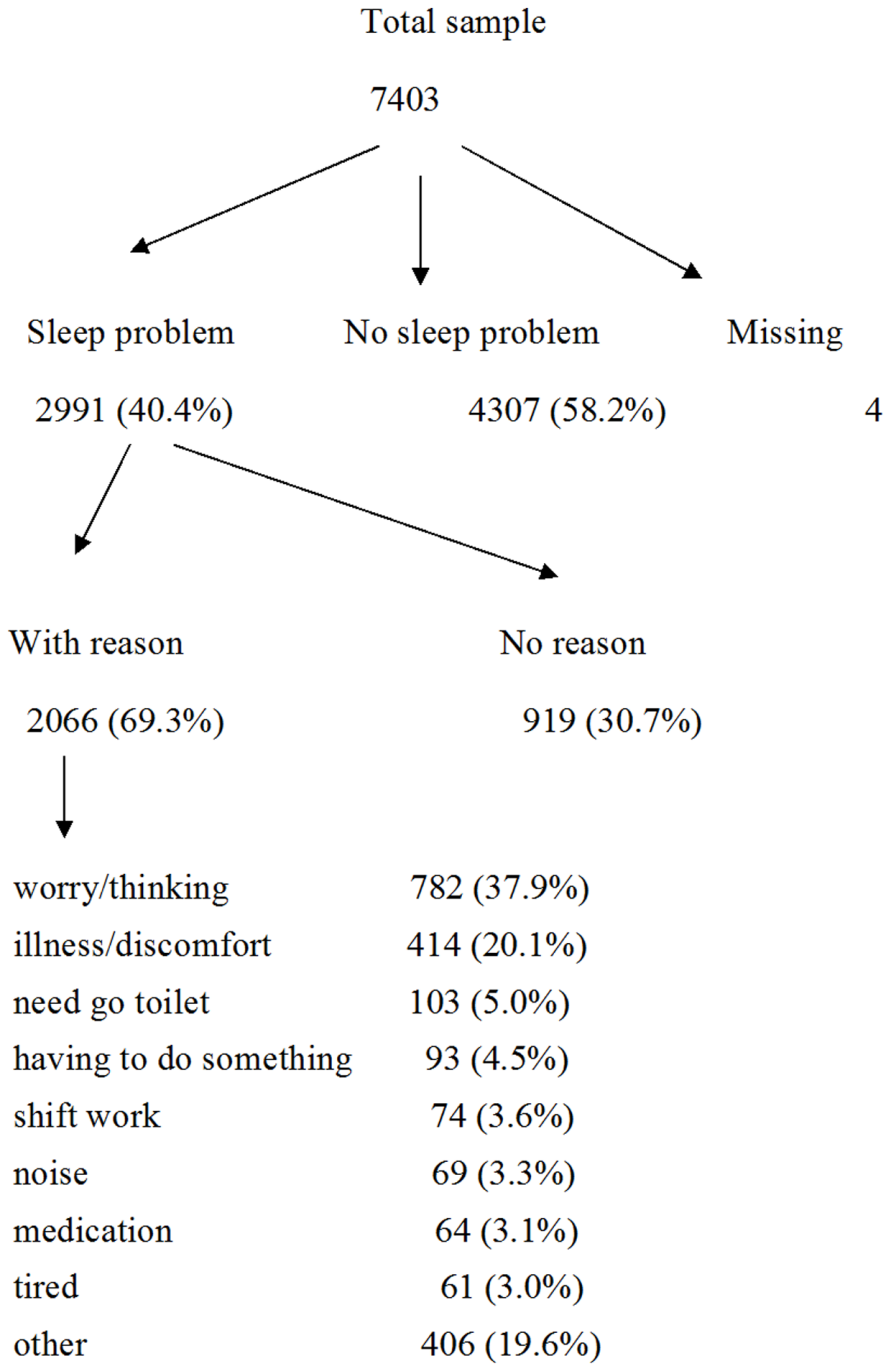
Diagram showing the number of people reporting sleep problems in the National Psychiatric Morbidity Survey and associated main reason.

Out of the 7,403 participants in the 2007 survey, 2,991 (40.4%) reported a problem sleeping in the previous week (Figure 1). Almost a third (31%) of those with sleep problems were unable to identify the main reason for their disturbance. Among the respondents (n = 2,066) who offered a specific reason for their sleep problems, worry/thinking (38%) was the most common, followed by illness/discomfort (20%) and ‘other’ (20%) reasons. A smaller proportion of respondents indicated going to the toilet (5%), having to do something (5%), shift work (4%), noise (3%), medication (3%), or tiredness (3%).

**Figure 2:**
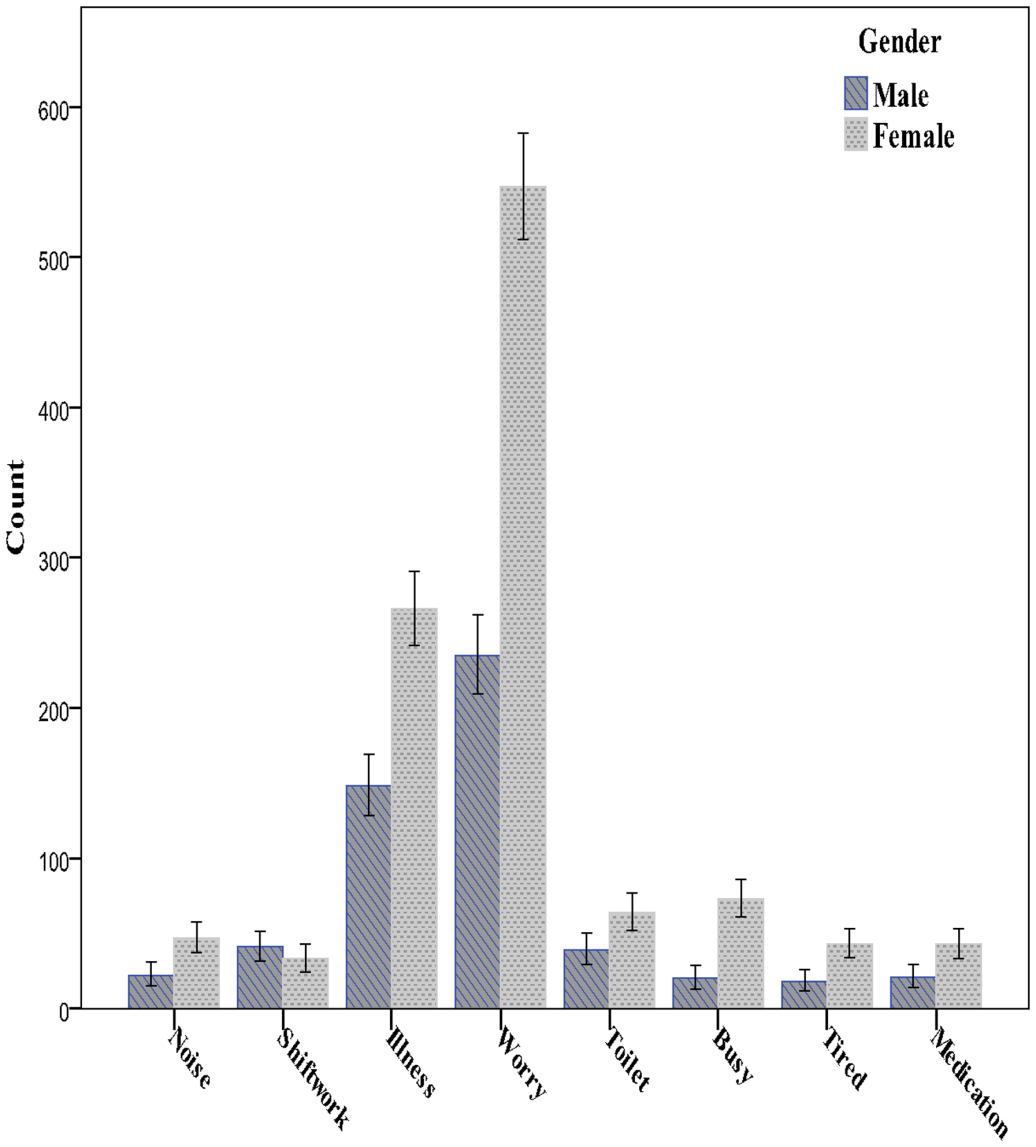
Reasons for sleep disturbance by gender. The bars represent the 95% upper 95% confidence intervals for the frequency of each reason for sleep problems by gender.

A significantly higher proportion of women (48.7%) compared with men (32.8%) reported problems with trying to get to sleep in the preceding month (chi square  = 167; df = 1; p<0.0001). These proportions were reflected in most of the reported reasons for poor sleep except for shift work (Figure 2).

**Figure 3:**
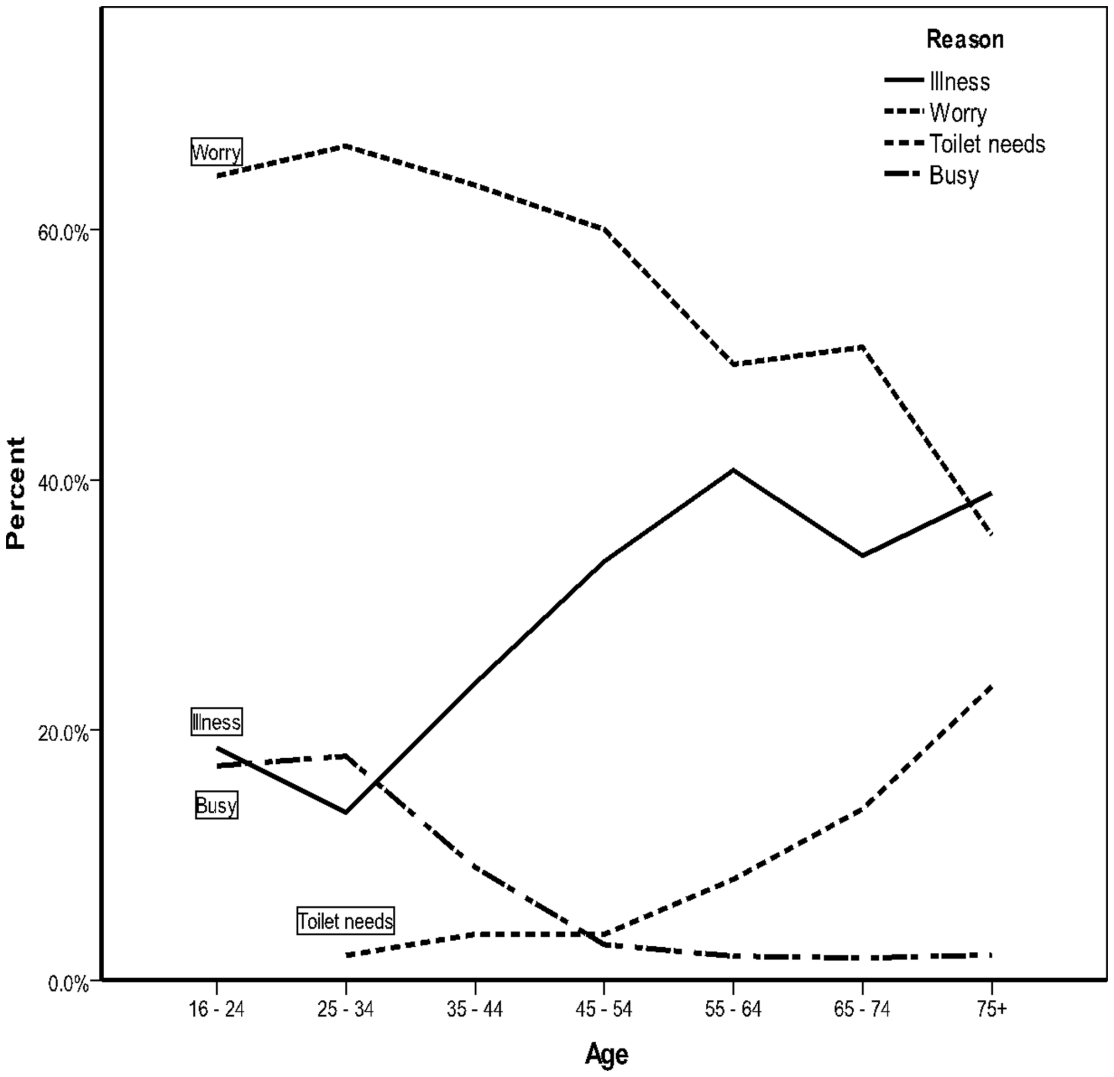
Distribution of main reasons for sleep problems by age groups.

Figure 3 shows the age distributions of the most common reasons provided by respondents. Different reasons showed different patterns: sleep problems through illness, for example, increased with age, plateauing in late middle age, while sleep disturbance through worry reached a peak in early working lives then declined.

**Figure 4:**
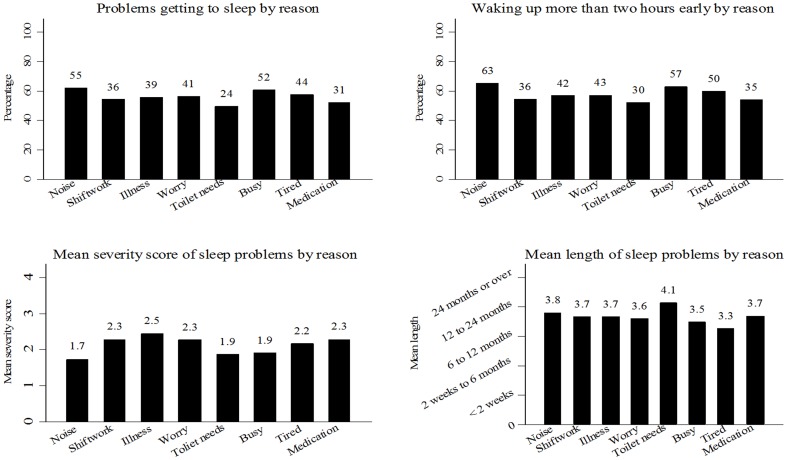
Graphs showing the patterning of the main reason for sleep problems with different sleep behavior measures.

The patterning of specific sleep disturbance symptoms is shown in Figure 4. The mean length of time sleep problems were reported did not vary markedly with reason, while problems getting to sleep, waking early and overall severity did show more, and often different, variability. Sleep disturbance because of illness, for example, was marked by the greatest severity but noise was most commonly associated with both early waking and delays in getting to sleep.

**Figure 5:**
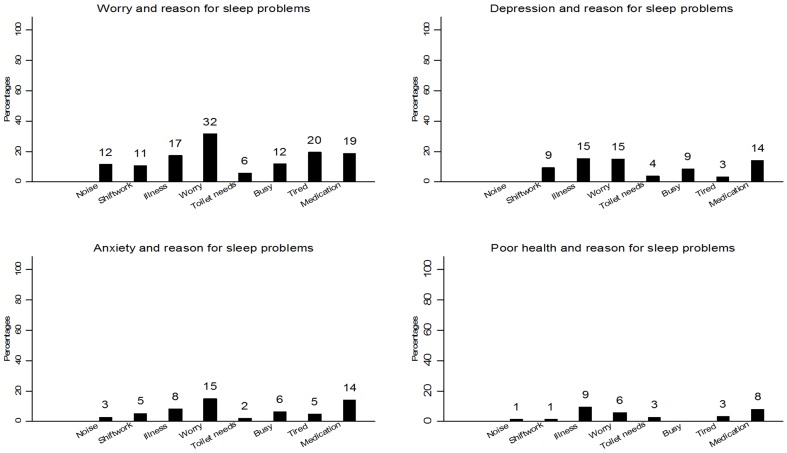
Graphs showing the patterning of the main reason for sleep problems with main sleep correlates.

Finally, Figures 5 shows the patterning of various correlates across the reasons for sleep disturbance. Those with poor self-reported health status were most likely to report problems sleeping, as might be expected, if the reason was illness or taking medicine but this group also scored highly on worry. Clinical depression was also related to sleep problems due to illness and medication but also doing shift-work and being busy.

## Discussion

The exact status of sleep disturbance or insomnia within medicine is unclear as it can be treated as a symptom or as a disease [13]. Indeed, DSM-IV separates ‘primary’ insomnia – which might be considered an illness in its own right – from ‘secondary’ insomnia in which the sleep problems are both consequence/symptoms of an underlying disease, such as mental illness, a general medical condition or medication. Such insomnia typologies rely on a medical model which separates symptom patterns which have no clear underlying ‘cause’ from those which appear to be brought about by underlying patho-physiological or psychological states. In this paradigm, participants' own views are at best simply another source of possible symptoms as they may be unaware of the underlying physiological and psychological processes which produce the problems they report.

Yet there are good grounds to be cautious in applying a biomedical paradigm to sleep disturbance. First, sleep problems only exist to the extent that they are reported by the patient: there are no independent corroborative ‘tests’ to determine the ‘objectivity’ of these subjective reports; indeed, the association between subjective and objective (as assessed in sleep laboratories) sleep is not very strong [14]. Sometimes, supposed underlying physical disease can be identified but how this affects sleep is often unclear. The patient's subjective report is therefore the main manifestation of insomnia and the association between this perception and supposed ‘objective’ underlying causes, when they can be identified, is not necessarily strong: in fact, current insomnia ‘types’ present a poor fit for reported symptoms [15]. The second grounds for being cautious about a biomedical interpretation of insomnia is that some ‘causes’ of sleep disturbance, such as noise, have little to do with medicine even though they may result in seeking medical treatments.

The other way of exploring insomnia types is to build on reasons rather than causes. Causes are the fundamental constructs which underpin biomedical science yet may be poor at explaining social cognition. Reasons on the other hand are part of the psychological narrative a patient uses to explain their illnesses. These may be the same as biomedical causes – but they may be different. This study therefore explored the value of using reasons as the underlying constructs in building an insomnia typology. A limitation of using the NPMS database for this purpose was that the range of reasons for sleep disturbance was pre-specified; nevertheless, the reasons the participants chose still give access to their own explanatory models rather than those used in past epidemiological research which, as noted, may have little personal meaning for participants.

The study used a national database which was primarily concerned with measuring psychiatric morbidity but as insomnia is such a common psychiatric symptom there were several sleep related questions in the survey. Prevalence figures were in line with other studies [16]–[18]. The fact that about one third of participants could not identify a reason for their insomnia does seem to be in accord with the proportion of insomnia categorised as ‘primary’ and may reflect similar classifications: both doctors and participants might share a failure to identify preceding states or events which resulted in insomnia. Equally many of the reasons respondents provided for their insomnia might reflect medical categories. Sleep disturbance due to illness and worry for example seem to correspond with the two major determinants of secondary insomnia, physical illness and anxiety/depression. The database used in the study allowed exploration of the latter hypothesis: are respondents who report sleep disturbance through worry those with psychiatric morbidity? Other studies have reported the close relationship between psychiatric morbidity and insomnia [19] but as shown in Figure 5 when respondents' reasons for sleep loss were plotted against anxiety, depression and worry as measured in the CIS-R, the correspondence between respondent and ‘medical’ diagnoses was not very strong.

The distribution of sleep disturbance by gender and age also show some divergent patterns to those usually assumed. Women tend to report more sleep problems than men in all age groups and while there have been suggestions that this might be explained by their different levels of anxiety and depression [20] this study suggests a more complex picture in which, for example, shift work was more likely to affect men. And while there was some increase with age for various reason groups this was certainly not for all; those losing sleep with ‘worry’ declined in number after an initial peak in the early working years.

The present study represents a cross-sectional analysis and it is possible that a current reason for sleep problems does not reflect a previous one. Further, longitudinal studies exploring within individual change in the reason for sleep problems would be of help. Also, the study is focused on people living in private households and the findings might not apply to people living in residential institutions. This issue does not invalidate the study findings, however, which apply to a nationally representative sample of adults living in England. An important strength of the study is that the wide age range of participants allowed investigation of reason for sleep problems across all sections of the adult population. An additional strength of the present study is the ability to explore the patterning of different reasons for sleep problems with its severity and duration, a phenomenon rarely explored in previous investigations.

This paper proposes a different way of thinking about sleep disturbance based on participants' own explanatory frameworks and seeing to what extent this alternative corresponds to the more usual biomedical or biopsychosocial perspective which include factors that may be outside of a participant's own world-view. Sleep problems, like many other patient symptoms, can have many causes and developing clearer typologies has been a common strategy in sleep medicine research. Using reasons to underpin a sleep nosology is an alternative way of sub-dividing participants' symptoms which has some face validity given the ‘subjective’ associations between reasons and symptoms. As this paper has tried to show, a typology based on reasons presents a different snapshot of the landscape of insomnia. In summary, while surveys of sleep disturbance in populations produce valuable estimates of overall prevalence they also probably hide many different types of problem. Teasing these different types out of the headline figures about how common this problem is in the population may enhance understanding of what might cause it, how it might manifest itself, and possibly, in the future, how different types might be clinically managed.
